# One organelle to rule them all: enhancement of lysosomal activity as a therapeutic modality for protein misfolding neurodegenerative diseases

**DOI:** 10.3389/fnins.2026.1941231

**Published:** 2026-09-23

**Authors:** Robert C. C. Mercer

**Affiliations:** Department of Biochemistry and Molecular Genetics, College of Graduate Studies, Midwestern University, Glendale, AZ, United States

**Keywords:** Aβ, lysosome, neurodegeneration, prion, tau, TDP-43, therapeutics, α-synuclein

## Abstract

Protein misfolding neurodegenerative diseases are invariably fatal conditions with a paucity of available and universally embraced disease modifying therapies. It is crucial, therefore, to identify additional potential candidate molecules and/or targets with which to work toward human trials. In this mini-review, I will highlight the recent discoveries of compounds that physically associate with the lysosome to enhance or restore its activity. One potential target for the development of additional therapeutics that emerges from the consideration of these molecules is the proton pump responsible for acidification of the lysosomal lumen, the vacuolar H^+^-ATPase. While the approach of directly targeting lysosomal activity remains experimental at present, the wealth of evidence pointing to the involvement of the endolysosomal-autophagic system across protein misfolding neurodegenerative diseases raises the tantalizing possibility that this therapeutic modality could have broad applicability.

## Introduction

Lysosomes, discovered by Christian de Duve in the 1950’s and so named in the pages of *Biochemical Journal*, were once thought to be little more than membranous sacs of hydrolytic enzymes (De Duve, 2005; De Duve et al., 1955). It is now understood that they play a central role in the coordinated destruction of damaged organelles, protein aggregates, and other unwanted cytoplasmic constituents through macroautophagy (referred to here as autophagy). During this process, intended cargo is sequestered in double-membrane autophagosomes that subsequently fuse with lysosomes, forming autolysosomes, to mediate the enzymatic degradation of their constituents (Nixon and Rubinsztein, 2024). Additional insight into lysosomal function has been provided by contemporary investigations that have identified these organelles as critical hubs for nutrient sensing and cellular signaling. For a comprehensive description of lysosomal biology, I direct readers to a recent authoritative review (Settembre and Perera, 2024). In this mini-review, I will describe several molecules that have been shown to enhance the proteolytic capacity of the lysosome through physical association and discuss their potential for the broad treatment of neurodegenerative disease. This will be prefaced with a brief introduction to the proteins with direct relevance to the mechanism of action of these compounds and a short description of the molecular pathologies common across protein misfolding neurodegenerative diseases.

## Lysosomal biology in brief

Mechanistic target of rapamycin complex 1 (mTORC1) plays a key role in the nutrient sensing capabilities of the cell, ultimately controlling the balance between catabolism and anabolism. In periods of abundance, amino acids generated by proteolysis are exported from the lysosomal lumen by dedicated transporters (Settembre and Perera, 2024). One of these, SLC38A9, functions as a guanine nucleotide exchange factor (GEF) to activate Rag-GTPases which then recruit mTORC1 to the lysosomal membrane through the formation of the Ragulator-Rag complex (Wang S. et al., 2015). Activation of mTORC1 promotes protein synthesis and inhibits protein catabolism through phosphorylation of downstream effectors. Important targets of mTORC1 include members of the MiT-TFE family of transcription factors, particularly transcription factor EB (TFEB), which has been described as the “master regulator of lysosomal biogenesis” (Settembre et al., 2011). Sequestered in the cytoplasm by mTORC1 phosphorylation, calcineurin dephosphorylation activates TFEB following lysosomal Ca^2+^ release, allowing for its translocation to the nucleus. There, TFEB is free to initiate the Coordinated Lysosomal Expression and Regulation (CLEAR) transcriptional program, which controls the expression of lysosomal hydrolases and membrane proteins, including numerous subunits of the vacuolar H^+^-adenosine triphosphatase (V-ATPase) (Settembre et al., 2011; Sardiello et al., 2009). The V-ATPase is responsible for maintaining the acidity of the lysosomal lumen (pH ~ 4.5), which is critical for the activity of the upwards of 60 acid hydrolases contained within (Xu and Ren, 2015). This multi-subunit proton pump is composed of a peripheral V_1_ domain with ATPase activity and a membrane spanning V_0_ domain that transports protons into the lysosomal lumen; dissociation of these domains renders the V-ATPase non-functional (Vasanthakumar and Rubinstein, 2020; Abbas et al., 2020). The V-ATPase also contributes to the association of mTORC1 with the lysosomal membrane through extensive interactions with Ragulator (Zoncu et al., 2011).

## Common molecular features of protein misfolding neurodegenerative diseases

Classical prion diseases are caused by the misfolding of the cellular prion protein (PrP^C^) into a disease associated conformation, PrP^Sc^, which, through self-templated propagation (Meisl et al., 2021; Gatdula et al., 2026a), accumulates in the central nervous system, causing widespread neuronal toxicity and death (Prusiner, 1998; Mercer and Harris, 2023). It is now generally appreciated that a “prion-like” propagation of aberrant protein structure is central to the molecular pathogenesis of other more common protein misfolding neurodegenerative diseases (Jaunmuktane and Brandner, 2020). The evidence for this is particularly strong when considering the molecular behavior of misfolded α-synuclein (Woerman et al., 2018; Woerman et al., 2015; Prusiner et al., 2015; Lau et al., 2020), Aβ (Stöhr et al., 2012; Watts and Prusiner, 2018; Jaunmuktane et al., 2015; Banerjee et al., 2024), tau (Woerman et al., 2016; Sanders et al., 2014; Kaufman et al., 2016), and transactive response DNA-binding protein 43 (TDP-43) (Nonaka and Hasegawa, 2018; Porta et al., 2021; Nonaka et al., 2013; Porta et al., 2018). Extending this concept, it has been proposed that at least some of these misfolded proteins should be classified as prions (Condello et al., 2024; Watts, 2019), though this designation remains controversial (Glynn et al., 2024).

Because neurons must maintain homeostasis over the lifetime of an organism, they are particularly susceptible to defects in lysosomal function which can, in and of themselves, lead to neurodegeneration (Udayar et al., 2022). The lysosome has been implicated in the pathobiology of numerous protein misfolding neurodegenerative diseases (Nixon and Rubinsztein, 2024) including prion disease (Laszlo et al., 1992; Kovács et al., 2007; Shim et al., 2016), Alzheimer’s disease (AD) and other tauopathies (Orr and Oddo, 2013; Lee et al., 2013; Piras et al., 2016), Parkinson’s disease (PD) and other synucleinopathies (Dilsizoglu Senol et al., 2021; Bellomo et al., 2020; Sahoo et al., 2022; Kakuda et al., 2024), and amyotrophic lateral sclerosis (ALS), a pathologically heterogeneous disease frequently associated with aggregates of TDP-43 (Wu Y. et al., 2021; Tanaka et al., 2023; Root et al., 2021; Chen et al., 2012). Further, mutations in lysosomal genes are causative of ALS (Akçimen et al., 2023; Brenner and Freischmidt, 2022) and PD (Klein and Mazzulli, 2018), and gene products involved in the endolysosomal system are known risk factors for AD (Szabo et al., 2022). The other mechanism of protein degradation, the ubiquitin-proteasome pathway, operates in the cytosol and terminates at large multi-subunit protein complexes known as proteasomes. This pathway has also been extensively investigated in the context of protein misfolding neurodegenerative diseases, but will not be discussed here (Church and Margolis, 2025; Arkinson et al., 2025).

## Broad therapeutic targeting of protein misfolding neurodegenerative diseases

There are examples in the literature of compounds reported to have neuroprotective activity against multiple pathologically misfolded proteins. The p38α MAPK inhibitor neflamapimod (VX-745) inhibits both PrP^Sc^ (Fang et al., 2018) and Aβ oligomer (Amin et al., 2019) mediated toxicity in assays using cultured hippocampal neurons. Chicago Sky Blue 6B has been reported to inhibit PrP^Sc^ propagation in cultured cells (Risse et al., 2015), reduce Aβ-mediated cognitive impairment *in vivo* (Yan et al., 2020), and decrease the aggregation and cell-to-cell propagation of α-synuclein (Min et al., 2022). Anle138b, which binds directly to misfolded protein species, has broad activity against PrP^Sc^ (Wagner et al., 2013), α-synuclein (Wagner et al., 2013; Heras-Garvin et al., 2019; Levin et al., 2014; Antonschmidt et al., 2022; Wegrzynowicz et al., 2019), Aβ (Martinez Martinez Hernandez et al., 2018; Han et al., 2025), and tau (Wagner et al., 2015). However, findings from classical prion disease offer a caution; Anle138b did not show efficacy in models of genetic prion disease (Vallabh et al., 2023; Qin et al., 2019) and it has been found to lead to the development of drug-resistant prions under certain conditions (Burke et al., 2020; Beauchemin et al., 2021). This phenomenon underscores the importance of prion strains (Block and Bartz, 2023; Manka et al., 2023) and their mutability when considering the therapeutic use of compounds that directly target misfolded protein species (Li et al., 2010; Oelschlegel and Weissmann, 2013; Berry et al., 2013; Ghaemmaghami et al., 2009).

Targeting cellular processes broadly important for the life cycle of pathologically misfolded proteins could bypass issues of strain biology and specificity. The induction of autophagy has received considerable attention in this regard, having been investigated as a therapeutic modality for prion disease (Abdelaziz et al., 2019; Heiseke et al., 2009; Aguib et al., 2009; Fan et al., 2015), tauopathies (Yang et al., 2022; Schaeffer et al., 2012), synucleinopathies (Lu et al., 2012; Wu et al., 2019), and ALS (Barmada et al., 2014). However, because autophagic flux requires functional lysosomes, increasing the rate of autophagy in a disease state where lysosomal function has been compromised could potentially cause more harm than good. Distinct from, but inextricably linked to, the induction of autophagy is the activation or restoration of lysosomal function, which has begun to emerge as a novel therapeutic strategy. Below, I will discuss several recent discoveries of compounds which have been shown to improve lysosomal pH and degrative capacity and highlight the effects of these compounds in cellular and animal models of neurodegeneration.

## Compounds that physically target the lysosome

### FK506

FK506 (tacrolimus), in addition to its widespread use as an immunosuppressant, is known to have neuroprotective and autophagy inducing properties (Rivera and Heitman, 2023). The V-ATPase catalytic subunit, ATP6V1A, was identified as a target of FK506 using the drug affinity responsive target stability (DARTS) assay (Kim et al., 2017). The authors went on to show that FK506 induces the nuclear translocation of TFEB, presumably through interruption of the V-ATPase/Ragulator-Rag complex (Kim et al., 2017) (Figure 1A). In experimental models of prion disease, FK506 treatment reduced the levels of PrP^Sc^ in cultured cells (Nakagaki et al., 2013) and modestly extended the life of prion inoculated mice (Nakagaki et al., 2020; Nakagaki et al., 2013). However, these effects upon prion disease pathogenesis have been challenged (Karapetyan et al., 2013). FK506 treatment has also been demonstrated to blunt the neurotoxicity induced by misfolded tau and α-synuclein; however, these effects have been ascribed to the compound’s inhibitory effects upon calcineurin (Scaduto et al., 2024; Marcatti et al., 2024; Zeng et al., 2024; Van Van der Perren et al., 2015; Caraveo et al., 2014), which should, paradoxically, prevent TFEB activation. Conversely, FK506 treatment was found to promote the pathological phosphorylation of TDP-43 and subsequent neurodegeneration in a *C. elegans* model of TDP-43 proteinopathy (Liachko et al., 2016).

**Figure 1:**
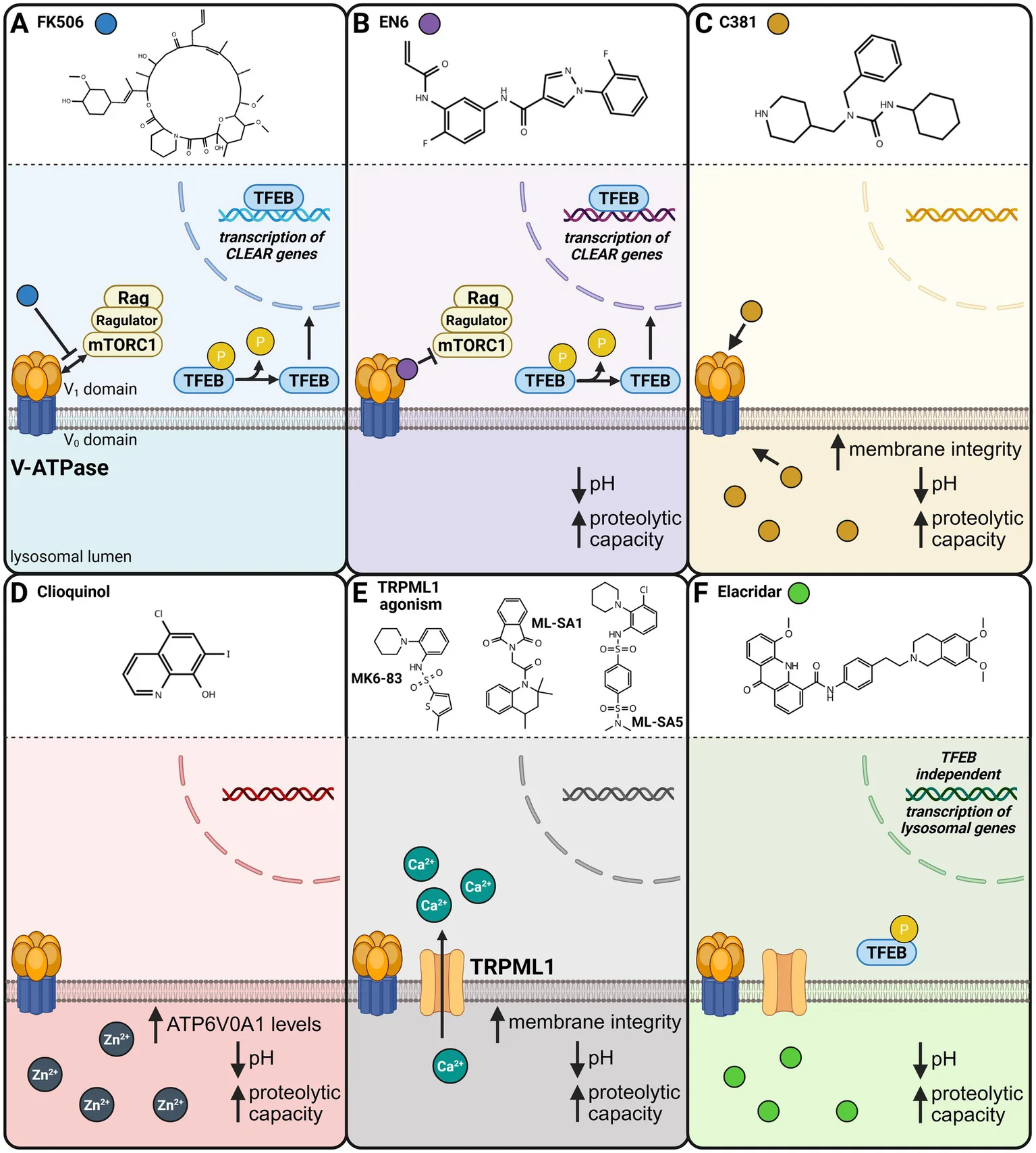
Mechanistic summary of lysosome activity-enhancing compounds. **(A)** FK506 binds ATP6V1A and inhibits recruitment of mTORC1 to the lysosomal membrane, resulting in TFEB activation and the induction of lysosomal biogenesis. **(B)** EN6 covalently binds to cysteine 277 of ATP6V1A, disrupting the interaction of the V-ATPase with the Ragulator-Rag complex and leading to the activation of TFEB. EN6 treatment decreases lysosomal pH and increases proteolytic capacity. **(C)** C381 accumulates within lysosomes where it decreases lysosomal pH, increases proteolytic capacity, and restores lysosomal membrane integrity. **(D)** Clioquinol, presumably through its ability to bind divalent cations, is proposed to increase Zn^2+^ levels within the lysosomal lumen. This leads to increased expression of ATP6V0A1, reduced lysosomal pH, and increased proteolytic capacity. **(E)** TRPML1 activation leads to the efflux of Ca^2+^ from the lysosomal lumen. This promotes the assembly of the V-ATPase and the subsequent decrease in lysosomal pH and increase in its degradative capacity. **(F)** Elacridar accumulates in lysosomes and promotes lysosomal gene expression, including *MCOLN1* (TRPML1) and subunits of the V-ATPase, through a TFEB-independent mechanism, leading to decreased lysosomal pH and increased proteolytic capacity. Figure created in BioRender. Mercer, R. (2026) https://BioRender.com/z7azgt1.

### EN6

EN6, a covalent activator of the V-ATPase, was discovered through a screen of cysteine and lysine reactive ligands using an autophagic flux assay (Chung et al., 2019). Chemoproteomics identified the target of EN6 to be cysteine 277 of the catalytic subunit of the V-ATPase, ATP6V1A (Chung et al., 2019). This modification was shown to prevent the recruitment of mTORC1 to the lysosome by inhibiting the physical association of the V-ATPase with the Ragulator-Rag complex (Zoncu et al., 2011; Chung et al., 2019), leading to the activation of TFEB and increased lysosomal acidification (Figure 1B). EN6 treatment significantly reduced the number of TDP-43 aggregates observed using an inducible cellular model; an effect demonstrated to be dependent on lysosomal activity by its abrogation using bafilomycin A1 (Chung et al., 2019). EN6 was further shown to be an effective inducer of autophagy *in vivo*, although examination was limited to skeletal muscle and cardiac tissue. It is currently unclear whether EN6 can penetrate the blood–brain barrier, or what safety concerns might be posed by irreversible modification of the V-ATPase over the long term. Further, EN6 is known to covalently attach to cysteine residues of other proteins, the effects of which have not been explored (Chung et al., 2019).

### C381

C381 (SRI-011381) is a known activator of TGF-β signaling whose molecular target was recently identified through a CRISPRi screen to be the V-ATPase (Vest et al., 2022). While the connection between TGF-β signaling and the V-ATPase is not well understood, C381, which is brain penetrant, has been shown to accumulate within lysosomes both *in vitro* and *in vivo*, promote lysosomal acidification and proteolytic activity, and reverse lysosomal membrane damage caused by L-leucyl-L-leucine methyl ester (LLOME) treatment (Chou et al., 2025; Vest et al., 2022) (Figure 1C). Using progranulin deficient mice to model frontotemporal dementia, a pathologically complex neurodegenerative disease in which tau and TDP-43 aggregates are often found (among others), C381 treatment was associated with reduced microgliosis, based on CD68 and Iba1 staining, and improved lysosomal function (Vest et al., 2022). Further, using the MPTP/probenecid model of PD, C381 protected dopaminergic neurons, improved cognitive and motor function, and reduced the accumulation of phosphorylated α-synuclein (Vest et al., 2022). In a follow-up study, C381 was shown to reduce the number of Aβ deposits in human cortical neurons transdifferentiated from fibroblasts isolated from AD patients (Chou et al., 2025). Another group demonstrated that treatment with C381 increased the phagocytosis of Aβ by a human microglial cell line and, using the 5XFAD model of AD, reduced the Aβ plaque burden and alleviated cognitive deficits (Liu et al., 2025). However, in this investigation improved phagocytosis and lysosomal destruction of Aβ was attributed to an increased expression of TMEM119, potentially through TGF-β activation, demonstrating that more work will be required to fully elucidate the effects of C381 (Liu et al., 2025). This mechanistic uncertainty also applies to positive effects of C381 treatment observed using models of traumatic brain injury (Li et al., 2024), multiple sclerosis (Wu Q. et al., 2021), and the demyelination phenotype observed in the microglial ablated *Fire^Δ/Δ^* mouse model (McNamara et al., 2023).

### Clioquinol

Clioquinol, an antifungal and antiprotozoal drug and divalent cation chelator, is also known to be an inducer of autophagy (Park et al., 2011). However, it has more recently been demonstrated that clioquinol treatment, through modulation of lysosomal Zn^2+^ levels, leads to an increase in the protein levels of ATP6V0A1, resulting in a decrease in lysosomal pH and promotion of protein degradation in primary cortical neuronal cultures (Wallings et al., 2019) (Figure 1D). Clioquinol has demonstrated neuroprotective properties in MPTP-induced mouse (Kaur et al., 2003) and primate (Shi et al., 2020) models of PD. In a transgenic mouse model expressing the A53T mutant of α-synuclein, clioquinol treatment improved cognitive and motor functions while also increasing the density of basal hippocampal dendritic spines (Finkelstein et al., 2016). Further, clioquinol was shown to alleviate dopaminergic neurodegeneration and α-synuclein pathology in transmissions of pathological human α-synuclein aggregates to mice (Teil et al., 2022) and improve cognitive performance and reduce Aβ plaque load in the CRND8 mouse model of AD (Grossi et al., 2009). Clinically, clioquinol is used topically but is not available for systemic administration due to a reported epidemiological association with the development of subacute myelo-optic neuropathy in Japan (Bareggi and Cornelli, 2012). Many of the studies described above did not directly assess the impact of clioquinol treatment on lysosomal biology. Nevertheless, further investigation of the influence of this molecule on lysosomes and its potential for the treatment of neurodegenerative diseases is warranted.

### TRPML1 channel agonists

MK6-83, ML-SA1, and the more potent ML-SA5, are agonists of the transient receptor potential mucolipin-1 (TRPML1) cation channel that is found in late endosomes and lysosomes and is important for proper lysosomal function and autophagic induction (Wang W. et al., 2015; Scotto Rosato et al., 2019; Bhattacharjee et al., 2024; Di Paola et al., 2018). Mislocalization of TRPML1 has been observed in prion infected cell cultures (Lakkaraju et al., 2021), and it has been found to be downregulated in the APP/PS1 transgenic mouse model of AD (Zhang et al., 2017). Agonism of these channels has been shown to activate lysosomal Ca^2+^ efflux (Tsunemi et al., 2019), promote assembly of the V-ATPase for maintenance of lysosomal acidification (Lee et al., 2025), increase the proteolytic rate of lysosomes (Qi et al., 2021), and promote lysosomal repair (Tan and Finkel, 2022) (Figure 1E). ML-SA1 has been shown to rescue the endolysosomal defects observed in iPSC derived neurons expressing APOE4, the strongest risk factor for the development of AD (Somogyi et al., 2023), and reduce intraneuronal deposits of Aβ in primary neurons from gp120/APP/PS1 transgenic mice (Bae et al., 2014). ML-SA5 has been reported to decrease the number of aggregates in cultures expressing a mutant form of α-synuclein (Pollmanns et al., 2022), and both ML-SA5 and MK6-83 were shown to reduce α-synuclein aggregation in iPSC derived neurons (Tsunemi et al., 2019). Supporting the agonistic targeting of TRPML1 for therapeutic intervention is the finding that its overexpression rescues the cognitive deficits observed in the APP/PS1 transgenic mouse model of AD (Zhang et al., 2017).

### Elacridar

Elacridar (GW120918) has been developed and extensively studied as a third-generation inhibitor of the MDR-1 and BCRP xenobiotic efflux pump and has generated clinical interest as an adjuvant in cancer chemotherapy (Dash et al., 2017). Transcriptomic analysis of elacridar treated cells revealed upregulation in genes encoding lysosomal hydrolases (including glucocerebrosidase (*Gba*), cathepsins A, B, and D, and β-galactosidase (*Glb1*)), membrane proteins (including *Mcoln1*, the gene encoding TRPML1), and multiple subunits of the V_0_ domain of the V-ATPase (*Atp6v0c*, *Atp6v0b*, *Atp6v0a1*, *Atp6v0e*, *Atp6ap1*) (Mercer et al., 2025). Consistent with these changes in gene expression, treatment with elacridar increased the fluorescence of both LysoTracker Red DND-99 and DQ Red BSA, reporter probes of acidity and proteolytic activity, respectively (Mercer et al., 2025). Elacridar, which accumulates in lysosomes, appears to act independently of TFEB nuclear translocation, distinguishing it from the other transcriptional activators discussed above and points to its influence upon an as-of-yet unidentified cellular pathway (Mercer et al., 2025) (Figure 1F). We were initially prompted to explore elacridar while investigating the anti-prion properties of σ receptor ligands (Mercer et al., 2024) and discovered that it is a potent anti-prion compound, able to prevent PrP-prion propagation and neurotoxicity at sub-micromolar concentrations and block the seeding activity of misfolded α-synuclein and tau in the micromolar range (Mercer et al., 2025). Ongoing research efforts are aimed at further elucidating the molecular mechanism of elacridar’s anti-prion effects and further characterizing its activity against misfolded α-synuclein and tau.

## Conclusion

While much remains to be explored, there is a growing body of evidence that the pharmacological restoration of lysosomal acidification and proteolytic capacity represents a tractable and broadly applicable therapeutic strategy for the treatment of protein misfolding neurodegenerative diseases (Nixon and Rubinsztein, 2024). Looking again to classical prion diseases, other currently explored therapeutic strategies involve lowering the production of PrP^C^ using RNAi (Gentile et al., 2026; White et al., 2008), antisense oligonucleotides (Raymond et al., 2019; Minikel et al., 2020; Cole et al., 2021), or epigenetic silencing (Neumann et al., 2024), blocking prion neurotoxicity by targeting downstream signaling cascades (Fang et al., 2018; Le et al., 2026; Mercer and Harris, 2019; Lin et al., 2025), and the use of dominant-negative inhibition of prion propagation (Gatdula et al., 2026b; Asante et al., 2015; Zerbes et al., 2026). While it will be of interest to ascertain the effectiveness of lysosomal enhancement as a stand-alone therapeutic approach, its use in combination with these other treatment strategies would be highly complementary. This approach might also permit the use of lower doses of lysosomal activity-enhancing compounds, potentially sidestepping issues of toxicity that could arise due to over acidification of the lysosome or the systemic modulation of the V-ATPase.

Many questions remain about the mechanism of action of the compounds described above and, in some instances, the evidence for their activity against pathologically misfolded protein species is weak. However, the common mechanistic theme that emerges from considering these molecules is the direct interaction with or the increased expression of V-ATPase subunits (Figure 1). The relevance of the V-ATPase to protein misfolding neurodegenerative disease is highlighted by a recent report demonstrating that overexpression of ATP6V0C rescued the lysosomal impairment induced by preformed fibrils of α-synuclein, leading to reduced cell-to-cell spread and increasing their degradation both *in vitro* and *in vivo* (Wang et al., 2026). Further, ATP6V1A has been identified through a multi-omics analysis as a key regulator of disrupted neuronal networks in late onset AD (Wang et al., 2021) and lysosomal acidification has recently been used as a readout in a drug screen against PD phenotypes (Tsukiboshi et al., 2026).

While this “one drug, many diseases” approach is attractive, it will be of critical importance to thoroughly investigate the consequences of targeting the V-ATPase, or lysosomal acidification more generally, in each neurodegenerative condition as the cellular mechanisms driving the propagation of these pathologically misfolded protein species are incompletely understood. Disease-stage and cell-type dependence, as well as the ability to penetrate the blood–brain barrier, are other important considerations that should be addressed before moving forward with a candidate molecule. Despite this word of caution, the studies described above and the growing appreciation for the role played by lysosomal dysfunction in the pathogenesis of neurodegenerative diseases (Colacurcio and Nixon, 2016; Song et al., 2020), point to the V-ATPase and lysosomal acidification as an exciting target with broad therapeutic implications.
